# Systemic Sonic Hedgehog Signaling Links Intestinal Nutrient Sensing With Sex‐Specific Type 2 Diabetes Progression

**DOI:** 10.1096/fj.202600261R

**Published:** 2026-03-19

**Authors:** Johannes Alfredsson, Nayere Taebnia, Najat Dzaki, Stefan A. Ljunggren, Ingela Helmfrid, Emilia C. Johansson, Jibbe Keulen, Olov Rolandsson, Ingvar Bergdahl, Volker M. Lauschke, Helen Karlsson, Mattias Alenius

**Affiliations:** ^1^ Department of Microbiology and Immunology Gothenburg University Gothenburg Sweden; ^2^ Department of Health, Medicine and Caring Sciences Linköping University Linköping Sweden; ^3^ Department of Physiology and Pharmacology and Center for Molecular Medicine Karolinska Institutet and University Hospital Stockholm Sweden; ^4^ Department of Molecular Biology Umeå University Umeå Sweden; ^5^ Dr Margarete Fischer‐Bosch Institute of Clinical Pharmacology Stuttgart Germany; ^6^ University of Tübingen Tübingen Germany; ^7^ Division of Micro‐ and Nanosystems Royal Institute of Technology (KTH) Stockholm Sweden; ^8^ Department of Public Health and Clinical Medicine Umeå University Umeå Sweden; ^9^ Department of Epidemiology and Global Health Umeå University Umeå Sweden; ^10^ Department of Pharmacy, the Second Xiangya Hospital Central South University Changsha China

**Keywords:** circulation, female, insulin resistance, organoids, plasma, prediabetes, sex differences, small intestine, Sonic Hedgehog, type 2 diabetes

## Abstract

Systemic Sonic Hedgehog (Shh) signaling is increasingly recognized as a potential regulator of adult metabolic homeostasis, yet its role in human disease remains poorly defined. In this study, we measured plasma Shh levels in two large Swedish cohorts to assess their associations with common non‐communicable diseases, with a particular focus on type 2 diabetes mellitus (T2DM) and metabolic dysfunction. A population‐based screen of 735 individuals revealed substantial inter‐individual variability in Shh levels, with elevated levels associated with T2DM and hypertension in females but not in males. These findings were validated in a nested case–control study, where Shh levels were significantly higher in T2DM females compared to matched controls. Correlation analyses showed that Shh levels were associated with insulin resistance in both sexes but reflected different disease states with early compensatory insulin secretion in males and late β‐cell dysfunction in females. Mechanistic studies using human intestinal organoids demonstrated that Shh secretion is induced by the combination of glucose and insulin, suggesting the intestine as a nutrient‐responsive source of the systemic Shh. Together, these results identify Shh as a sexually dimorphic marker of metabolic dysfunction and support its functional role in glycemic control and metabolic disease.

## Graphical abstract

Here, Systemic Sonic Hedgehog (Shh) emerges as a sex‐specific metabolic signal and potential biomarker. Across two large cohorts, Shh rose selectively in females with type 2 diabetes, with a sharp pre‐diagnostic increase in about one‐quarter of cases, indicating value for early risk stratification. Mechanistic intestinal organoid studies show that, as in *Drosophila*, combined glucose and insulin stimulate Shh secretion, revealing a conserved gut–endocrine axis with a putative relevance for predicting and managing type 2 diabetes in females.

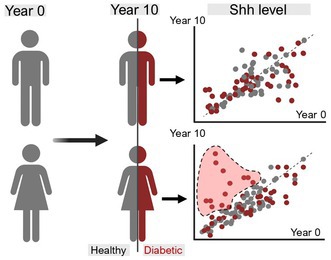

## 1 Introduction

The Hedgehog (Hh) pathway is a highly conserved signaling mechanism that governs embryonic development and tissue patterning across metazoans [1]. Originally identified in *Drosophila melanogaster* , the core components and functions of the Hh pathway have remained remarkably preserved throughout evolution [2, 3], underscoring its critical biological functions.

In vertebrates, the pathway is mediated by three paralogous ligands, Sonic Hedgehog (Shh), Indian Hedgehog (Ihh), and Desert Hedgehog (Dhh), with Shh being the most extensively characterized [1, 2]. During embryogenesis, Shh signaling orchestrates a wide array of developmental processes, including axis formation, organogenesis, and the specification of cell fate [1, 2, 3]. In the adult organism, the pathway continues to play essential roles in the regulation of stem cell maintenance, tissue regeneration, and homeostasis [4, 5].

The investigation of Shh function in adult tissues has been complicated by its indispensable role during development, which limits the use of conventional genetic models for functional interrogations. Nevertheless, recent studies in *Drosophila* have shown that Hh signaling can function as an important metabolic cue in adults [6, 7, 8, 9]. In this context, Hh is produced in the gut and, upon high dietary sugar intake, is released into the circulation where it is transported via lipoprotein particles [9, 10, 11]. Circulating Hh in flies modulates feeding behavior by suppressing sweet taste perception and shifting dietary preference from sugars to fats [6, 9]. These findings suggest that systemic Hh acts as a proxy signal for sugar feeding history and plays a role in balancing metabolism and nutrient intake.

In mammals, including humans, Shh is similarly transported in lipoprotein particles [8, 11, 12, 13], indicating that systemic Hh signaling is evolutionarily conserved. In the human small intestine, Shh is expressed in the epithelial cells and signals to the surrounding mesenchyme [14, 15]. However, the origin of circulating Shh in humans remains unclear. Small clinical studies in selected patient groups [16, 17, 18] have reported altered plasma Shh levels in various non‐communicable diseases, including cancers, autism, and type 2 diabetes mellitus (T2DM), suggesting that systemic Shh may serve as a biomarker for disease onset or progression. Importantly, while metabolic diseases such as T2DM and hypertension exhibit well‐documented sex differences in prevalence, progression, and treatment outcomes, stratified analyses into sex‐specific patterns of Shh levels have not been reported.

In this study, we conducted a comprehensive screen of plasma Shh levels in a large Swedish cross‐sectional cohort to systematically examine the relationship between Shh levels and the 12 most prevalent non‐communicable diseases in both sexes. Notably, we identified significant correlations between elevated plasma Shh levels and T2DM as well as hypertension in females, but not in males. We validate our findings in a second independent cohort, which included longitudinal clinical data, thereby enabling us to track Shh plasma dynamics in relation to T2DM progression. Additionally, we performed mechanistic experiments using human intestinal organoids to identify nutrient and hormonal regulation of Shh expression and secretion in patient‐derived cultures. Our results identify intestinal enterocytes as a glucose‐ and insulin‐responsive source of systemic Shh also in humans. Combined, our data suggest that Shh provides an important mediator that links intestinal nutrient levels to glycemic control and the development of T2DM.

## 2 Research Design and Methods

### 2.1 The Glass Works Cohort, a Regional Cross‐Sectional Cohort

The study population comprised inhabitants from a restricted area, a village with an active glass manufacturing activity in south/central Sweden [19]. A total of 735 individuals had a blood sample drawn and these samples were provided to us by the author of the original article. Questionnaire data indicate that most participants had their blood drawn 2–3 h after their last meal. The cohort is sex balanced and show age‐skewing toward an older population. For creating disease groups and identifying a healthy cohort, we used questionnaire data consisting of both closed and open (free text) questions. The closed questions were as follows: Diabetes (Yes/No), Epilepsy (Y/N), Hypertension (Y/N), Cardiovascular disease (Y/N), Stroke (Y/N), Kidney disease (Y/N), Memory disturbance (Y/N), Pulmonary disease (Y/N), Other disease (Y/M), History of Cancer (Y/N), Medicates regularly (Y/N). Free‐text responses were used to identify the following conditions: allergies/asthma, coronary artery disease (cardiovascular disease from closed questions plus specifying angina pectoris or myocardial infarction in free‐text), inflammatory skin disorders (e.g., psoriatic disease, eczema, or allergic contact dermatitis), inflammatory bowel disease (ulcerative colitis or Crohn's disease), rheumatic diseases (e.g., rheumatoid arthritis, ankylosing spondylitis, psoriatic arthritis, polymyalgia rheumatica), and thyroid disorders (inferred from self‐reported thyroid hormone replacement therapy). 217 individuals reported negative on all closed questions and to any type of medication and were therefore defined as the healthy control group.

### 2.2 Plasma Sonic Hedgehog Analysis

N‐terminal Sonic Hedgehog levels in plasma and culture medium were quantified using a commercial Human ShhN ELISA kit from Sigma‐Aldrich. The ELISAs were performed according to the manufacturer's protocol (with incubations of 2.5 h at room temperature or overnight at 4°C). Every case/control pair in the VIP study was analyzed on the same ELISA plate to minimize inter‐assay variance. Absorbance was measured at 450 nm on a FLUOstar plate reader (BMG LabTechnologies). The ClarioStar software was used to generate a standard curve and calculate individual values. As plasma samples from both cohorts had been previously thawed, we conducted a stability control experiment to assess potential degradation of Shh. The results demonstrated that Shh protein levels remained stable for at least 18 h at 4°C and up to 6 h at room temperature (0 Hr 9258.00, SD = 198.93; 18 Hr, 9162.33, SD = 207.89; 6Hr, 9213.67, SD = 58.93), indicating that Shh is extremely stable in plasma. We monitored intra‐ and inter‐plate coefficients of variation (CVs), which remained within acceptable limits (< 10%).

### 2.3 A T2DM Case–Control Cohort Within VIP

Prospectively collected plasma samples from the Västerbotten Intervention Programme (VIP) sub‐cohort within the Northern Sweden Health and Disease Study were obtained [20, 21]. T2DM had been identified by the Diabetes Register in Northern Sweden [22] and studied in a previous study [20, 21]. The present study used samples from 135 T2DM cases and an equal number of sex‐ and age‐matched controls. We only included individuals who had both a sample at baseline, prior to a T2DM diagnosis, and one 10 years later, after having a T2DM diagnosis.

The values for cases and controls were pooled for the correlation analyses between Shh and the various metabolic markers in Figure 5. HOMA2 Calculator v2.2.3 (Diabetes Trials Unit, University of Oxford) was used for calculating HOMA2‐%B, HOMA2‐%S, and HOMA2‐IR using fasting glucose (mmol/L) and C‐peptide (nmol/L), determined by routine methods at Linköping University Hospital in Linköping, Sweden.

### 2.4 Human Intestinal Organoid Culture and Differentiation

Human intestinal epithelial organoids were established from jejunal tissue samples as described elsewhere [23]. The procedures were approved by the national governing body (Etikprövningsmyndigheten, Sweden, permit 2023–01524‐01) and followed informed written consent. Culture samples were fully anonymized prior to processing and experimentation. Handling of patient‐derived cells was approved under permit numbers 2024‐05808‐01.

Cryopreserved human jejunal organoids were thawed rapidly and transferred to ice‐cold DMEM/F12 containing 0.25% bovine serum albumin (BSA), and centrifuged. Pellets were resuspended in complete IntestiCult Organoid Growth Medium (STEMCELL Technologies) supplemented with penicillin–streptomycin, embedded in ice‐cold Matrigel (Corning), and seeded as 50 μL domes in pre‐warmed 24‐well plates. After polymerization at 37°C, 500 μL of complete growth medium containing 10 μM ROCK inhibitor (Y‐27632) was added to each well. Medium was replaced with Y‐27632–free growth medium on day 3, and cultures were passaged every 7–10 days at a split ratio of ~1:6–1:8 using Gentle Dissociation Reagent (STEMCELL Technologies).

For differentiation, medium‐sized, high‐confluence organoids were released from Matrigel using Cell Recovery Solution (Corning) on ice for 60–90 min with gentle agitation, washed in ice‐cold Williams E medium (Pan‐Biotech) supplemented with 0.25% BSA, and seeded in ultra‐low attachment 24‐well plates (Corning) at ~500 μL/well in custom differentiation medium. This consisted of Williams E (without glucose, phenol red) supplemented with 5.5 mM glucose, 1× GlutaMAX, 1× penicillin–streptomycin, 10 mM HEPES, 1× B27 supplement minus insulin, 1 mM N‐acetylcysteine, 0.58 ng/mL recombinant human insulin, 10 nM [Leu15]‐gastrin, 50 ng/mL recombinant mouse EGF, 100 ng/mL recombinant mouse Noggin, 500 nM A83‐01, 10 μM DAPT, and 2 μM IWP‐2. Cultures were maintained at 37°C, 5% CO_2_ for 3 days without medium change before experimental treatments.

For nutrient–hormone stimulation assays, differentiated organoids were exposed to combinations of glucose (low, 5.5 mM; medium, 11 mM; and high, 22 mM) and insulin (physiological low, 0.1 nM; high, 1.7 μM) in Williams E base medium without differentiation growth factors for 48 h. Organoid lysates were collected for RNA extraction, and conditioned media were harvested for Shh quantification by ELISA.

### 2.5 Quantitative PCR Analysis

Total RNA was extracted using the Quick‐RNA Micro Kit (Zymo Research) according to the manufacturer's protocol, including the in‐column DNase I digestion step to remove residual genomic DNA. RNA concentration and purity were assessed using a NanoDrop spectrophotometer (Thermo Fisher Scientific). For each sample, ~100 ng total RNA was reverse transcribed into cDNA. Quantitative PCR (qPCR) was performed on a StepOne Real‐Time PCR System (Applied Biosystems) using TaqMan Gene Expression Assays (Applied Biosystems) with TaqMan Universal PCR Master Mix. Each reaction was run in duplicate in a 20 μL volume. Expression of *SHH* was normalized to the endogenous control gene *GAPDH*, and relative transcript levels were calculated using the ΔΔCt method. The TaqMan assay IDs for *SHH* and *GAPDH* are hs00179843_m1 and hs99999905_m1, respectively.

### 2.6 Statistical Methods

Non‐parametric methods were used for all analyses involving Shh on the original scale: the Mann–Whitney U test for comparing two independent groups and Spearman's Rank‐Order Correlation for assessing correlations. Since individuals with self‐reported diseases may belong to multiple disease groups, comparisons between each disease group and a healthy reference were independently assessed using the Mann–Whitney *U* test, with *p*‐values adjusted for multiple comparisons using the Bonferroni method. For visualization in scatterplots of age against Log_10_ Shh, linear regression was used to fit trend lines, complementing the Spearman's correlation analysis. All statistical analyses and graphs were conducted using GraphPad Prism 10.4.0, with a significance level set at *p <* 0.05.

### 2.7 Data and Resource Availability

Data and resources are available on reasonable request.

## 3 Results

### 3.1 Large Inter‐Individual Variation of Plasma Shh Levels

Given the emerging evidence that systemic Hh signaling may reflect metabolic state [9] and contribute to disease processes, we investigated whether circulating Shh levels are also associated with common non‐communicable diseases in humans. To this end, we conducted a comprehensive screen of plasma Shh levels in a large, population‐based Swedish cohort comprising plasma samples from 735 individuals (351 males and 389 females) with an average age of 60 years (SD = 13.7) and a mean body mass index (BMI) of 26.1 kg/m^2^ (see Table S1 for details).

**FIGURE 1:**
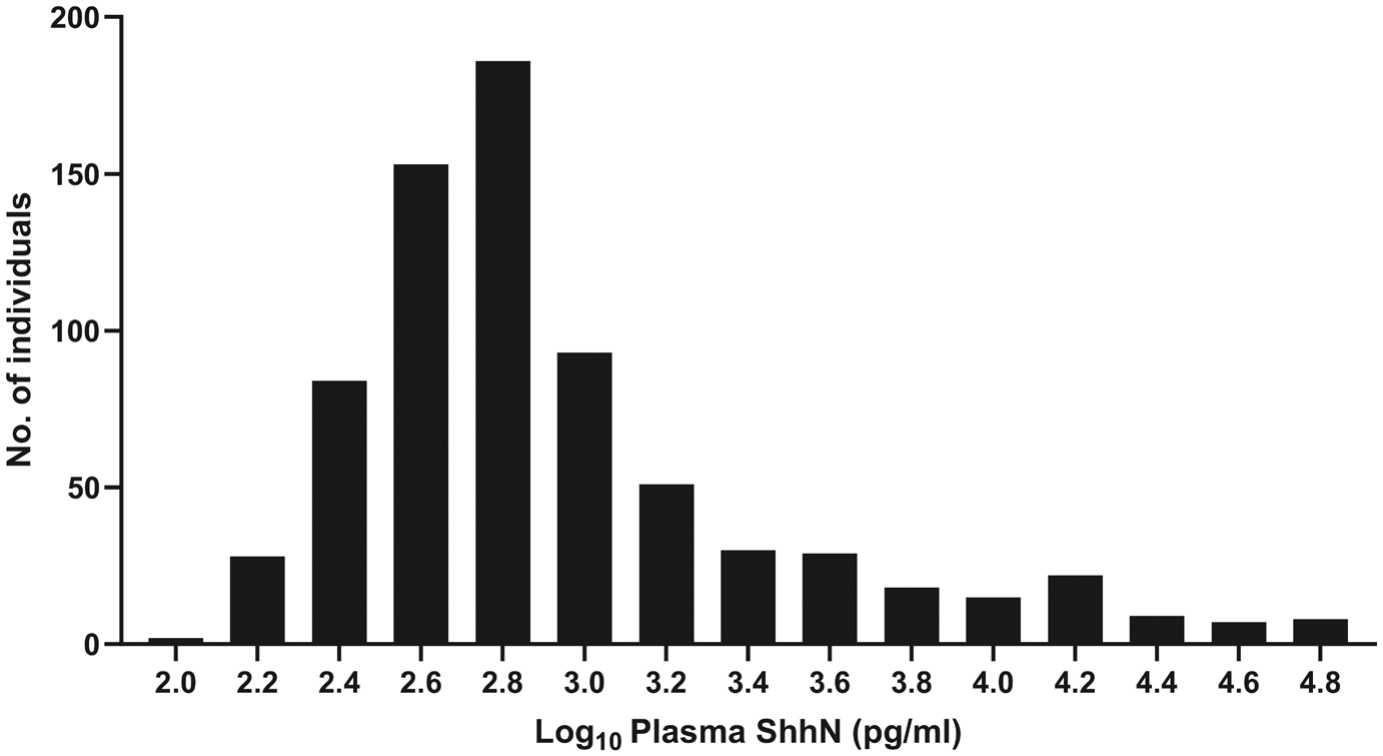
Plasma Shh levels differ substantially between individuals. Histogram shows log‐transformed plasma Shh levels for the 735 individuals in the cross‐sectional cohort. The skewed distribution highlights the large variability in plasma Shh levels observed between individuals.

ELISA analysis of the samples from the whole cohort showed an extensive interindividual variability in plasma Shh levels that spanned nearly three orders of magnitude (100–68 720 pg/mL; median 627 pg/mL; Figure 1). This wide distribution is consistent with previous reports showing plasma Shh levels spanning from a few pg/mL to hundreds of ng/mL [16, 17, 18, 24, 25, 26]. The distribution was also markedly tilted; while the first three quartiles varied tenfold (100–1311 pg/mL), the upper quartile spanned sixtyfold (1311–68 720 pg/mL), suggesting that factors such as sex, age, or disease status may contribute to elevated Shh levels.

### 3.2 Systemic Shh Levels Correlate With Sex

**FIGURE 2:**
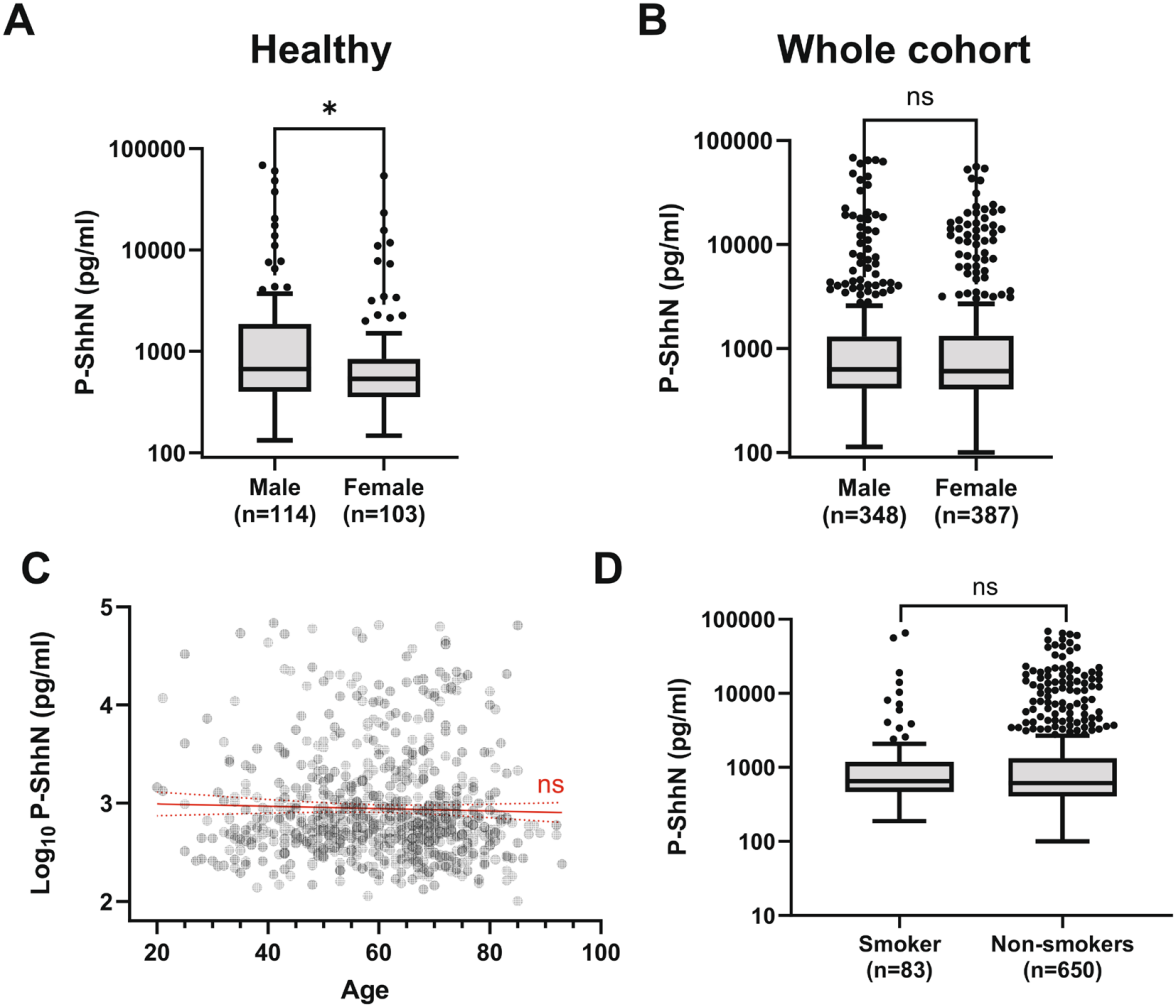
Sex difference in basal Shh levels. (A) Boxplot comparing plasma ShhN levels between healthy males and females. Median ShhN levels were significantly lower in females. (B) Boxplot comparing Shh levels between males and females in whole cohort. (C) Scatter plot of Log10‐transformed Shh plasma levels versus age. The red line shows the linear regression fit with a 95% confidence interval. (D) Boxplot comparing Shh levels between smokers and non‐smokers. *p*‐values. ns = non‐significant; *p < 0.05.

To assess the relationship between Shh levels and demographic factors, we first analyzed Shh levels in 103 females and 114 males reporting no disease or medication use. Within this cohort, healthy males had significantly higher plasma Shh levels than healthy females (Mann–Whitney *U* test, *p* = 0.016, Figure 2A). Notably, when including individuals with self‐reported disease history of 12 clinical categories of common non‐communicable disorders, this sex difference was not observed in the full cohort (Mann–Whitney *U* test, *p* = 0.66, Figure 2B), suggesting that Shh levels in females may be more sensitive to disease‐related changes.

In contrast to sex, our analysis showed no significant age‐related variation in Shh levels (Slope [95% CI] = 0.001 [−0.004 to 0.002], *p* = 0.411, Spearman's correlation: *r* = −0.03, *p* = 0.355, Figure 2C), contrary to a previous study reporting an inverse correlation between Shh levels and age [26]. Similarly, we found no association between smoking status and plasma Shh concentrations (Mann–Whitney *U*, *p* = 0.723, Figure 2D), indicating that other factors, such as sex or disease, may underlie the observed skewing.

### 3.3 Elevated Plasma Shh Levels Associate With Type 2 Diabetes and Hypertension in Females but Not in Males

**FIGURE 3:**
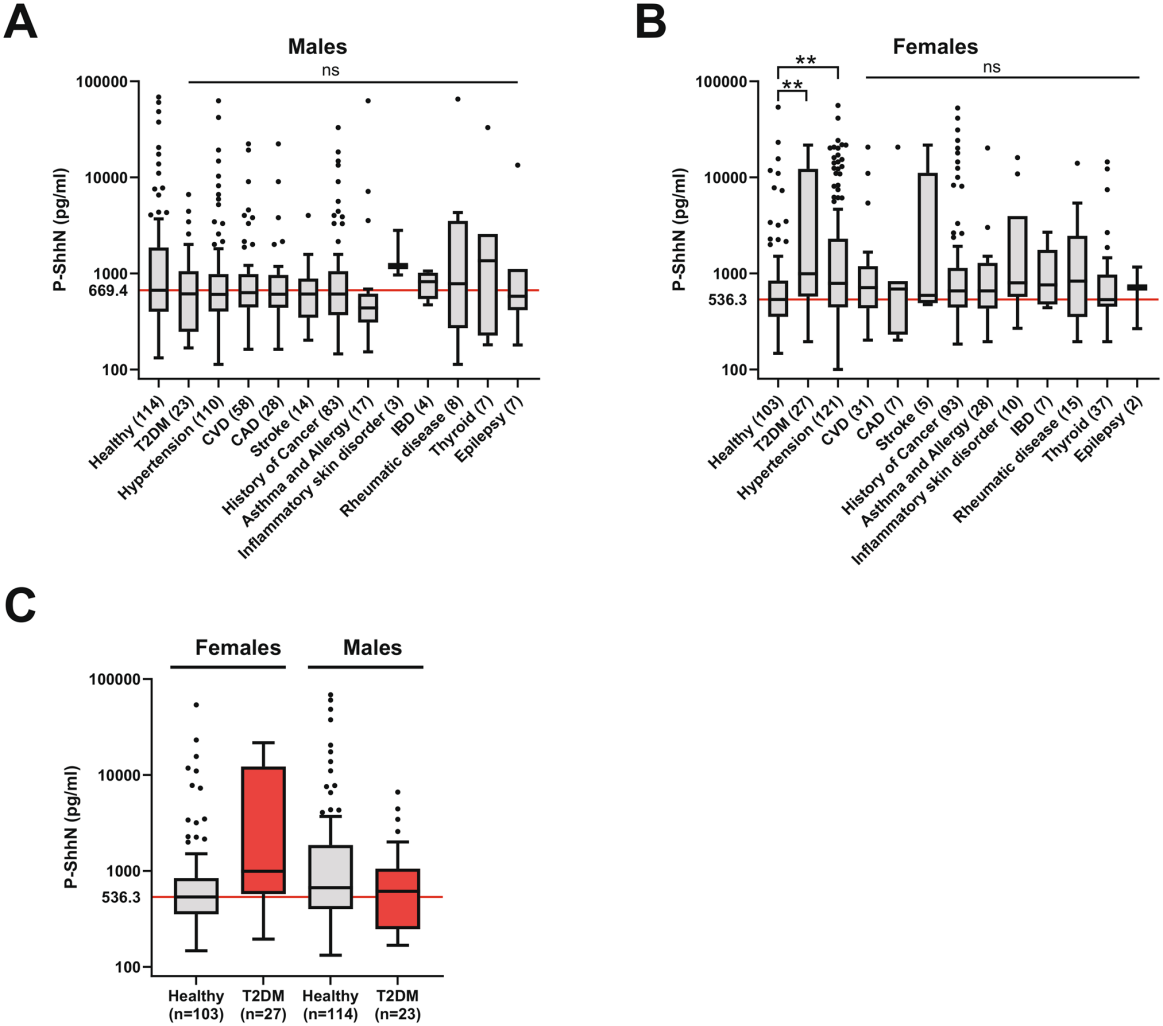
Plasma Shh levels relate to T2DM and hypertension in females. (A–C) Comparison of plasma Shh values of 12 different diseases groups versus the healthy control group. (A) In females, the hypertension (adj. *p* = 0.007) and diabetes (adj. *p* = 0.023) groups show significant increased plasma Shh levels compared to healthy females. (B) In males, no disease group showed significant differences compared to the control group. (C) Boxplots show plasma Shh levels for the control and type 2 diabetes groups. The red line indicates the median Shh level in healthy females. Statistical significance was assessed using the Mann–Whitney *U* test and Bonferroni correction was used to adjust obtained *p*‐values for multiple comparisons. ns = non‐significant; *Adj. *p* < 0.05; **Adj. *p* < 0.01.

To address if Shh levels are sensitive to disease‐related changes, we evaluated associations between disease groups and the healthy cohort stratified by sex (Table S2). Critically, we found significantly elevated Shh levels in females with T2DM (adj. *p* = 0.007) and hypertension (adj. *p* = 0.023, Figure 3A) compared with healthy controls. In contrast, no disease category showed significant Shh elevation when compared to the healthy group in males (adj. *p* > 0.05; Figure 3B). Further comparison between male and female T2DM patients revealed that females had both lower pre‐diagnosis baseline Shh levels and higher levels post‐diagnosis (Figure 3C), suggesting a stronger Shh response in female T2DM progression.

### 3.4 Validation of Shh–T2DM Association in an Independent Cohort

**FIGURE 4:**
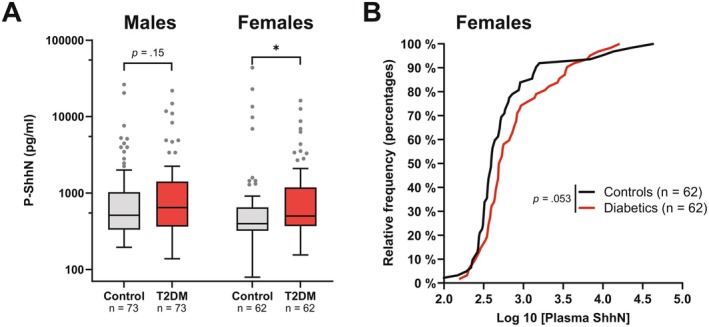
Elevated plasma Shh levels are associated with T2DM in females. (A) Boxplot comparing the plasma ShhN levels in the type 2 diabetes cohort versus age and sex‐matched controls grouped by sex. Median levels were significantly higher for female diabetics versus controls (*p* = 0.026 Mann–Whitney *U*). (B) Cumulative Kolmogorov–Smirnov frequency distribution of Shh values for the female panel shown in ‘A’.

To validate the observed association between elevated plasma Shh levels and T2DM, we performed a nested case–control study within a second, independent cohort, the Swedish Västerbotten Intervention Program (VIP; Table S3) [20, 21]. This cohort included 73 males and 62 females diagnosed with T2DM at or prior to the time of sampling, each with a sex‐ and age‐matched healthy control. Consistent with our initial findings and previous research [26], females with T2DM showed significantly elevated Shh levels (501 pg/mL, IQR: 369–1187 pg/mL) compared to their healthy counterparts (398 pg/mL, IQR: 320–649 pg/mL, *p* = 0.03; Figure 4A), supporting an increase in Shh levels, specifically in female T2DM patients. In contrast, no significant difference in Shh levels was observed between male T2DM cases (median = 513 pg/mL, IQR: 331–1026 pg/mL) and healthy male controls (median = 646 pg/mL, IQR: 363–1413 pg/mL; *p* = 0.15, Figure 4A). To assess whether T2DM contributes to the observed elevation in Shh plasma levels in females, we compared distribution curves between diabetic and healthy females (Figure 4B). Interestingly, the curves overlapped at the extremes but diverged considerably between the 50th and 90th percentiles, suggesting that factors other than T2DM drive Shh outlier values whereas the T2DM‐associated increase in females rather appears at intermediate Shh levels.

Taken together, these findings from the two independent cohorts demonstrate that elevated plasma Shh levels are associated with T2DM in females but not in males, suggesting a sexually dimorphic role for systemic Shh signaling in metabolic disease.

### 3.5 Shh Levels Associate With Metabolic Parameters and Disease Phenotypes in a Sex‐Specific Manner

**FIGURE 5:**
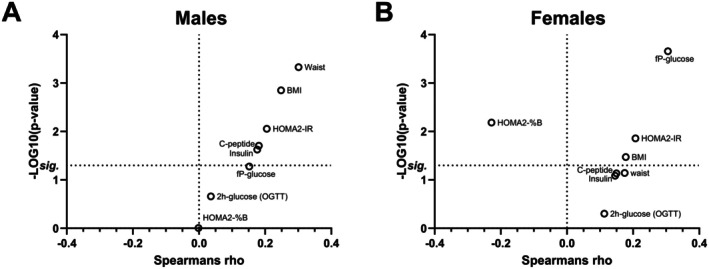
Sex differences in the relationship of Shh levels to various metabolic parameters and obesity. Plotting Spearman's rank correlation coefficient against the negative log10 *p*‐value for correlations between plasma Shh levels and diabetic parameters in males and females. The horizontal dotted lines labeled “sig” denotes statistical significance (*p* = 0.05).

Next, we analyzed associations between circulating Shh levels and clinical markers of metabolic dysfunction (Table S4). Notably, Shh levels were significantly correlated with insulin resistance, as measured by the HOMA2‐IR index in both males and females (Figure 5A,B). This finding indicates that systemic Shh levels rise in individuals with impaired insulin sensitivity, independent of sex.

We further analyzed the relationship between Shh and metabolic markers across different T2DM stages. Interestingly, we found clear sex‐specific differences in the correlations with these markers. In males, Shh levels were positively correlated with fasting insulin and C‐peptide concentrations (Figure 5A), reflecting increased insulin secretion by pancreatic β‐cells, a compensatory response typically seen in prediabetes [27]. In females, however, Shh levels were inversely correlated with HOMA2‐%B (Figure 5B), a measure of β‐cell function that estimates the ability of β‐cells to produce insulin relative to glucose levels. Additionally, Shh levels in females with T2DM were positively correlated with fasting plasma glucose (fP‐glucose, Figure 5B), together indicating a late prediabetes induction of Shh in females.

Given the well‐established link between obesity and increased risk of T2DM [28], we next examined associations between Shh levels and anthropometric measures of adiposity (body mass index (BMI) and waist circumference). In males, Shh levels showed a moderate positive correlation with both BMI (Spearman's *ρ* = 0.25, *p* = 0.001) and waist circumference (*ρ* = 0.30, *p* = 0.0005) (Figure 5A), whereas in females, Shh levels showed only a weak correlation with BMI (*ρ* = 0.18, *p* = 0.03) and no significant correlation with waist circumference (Figure 5B). These observations are consistent with Shh levels in females being associated with later‐stage T2DM markers and thus align with previous reports that obese females are more likely to remain metabolically healthy and normoglycemic compared to obese males [28].

Together, these findings show that elevations in Shh levels are paralleled by increases in insulin resistance and hyperglycemia, but the inflection point at which Shh levels change differs between sexes. In males, elevated Shh are associated with obesity and may signal compensatory insulin secretion, characteristic of prediabetes, while in females, it appears to be associated with declining β‐cell function and worsening glycemic control later in disease progression.

### 3.6 Longitudinal Stability and Disease‐Associated Elevation of Plasma Shh in T2DM

**FIGURE 6:**
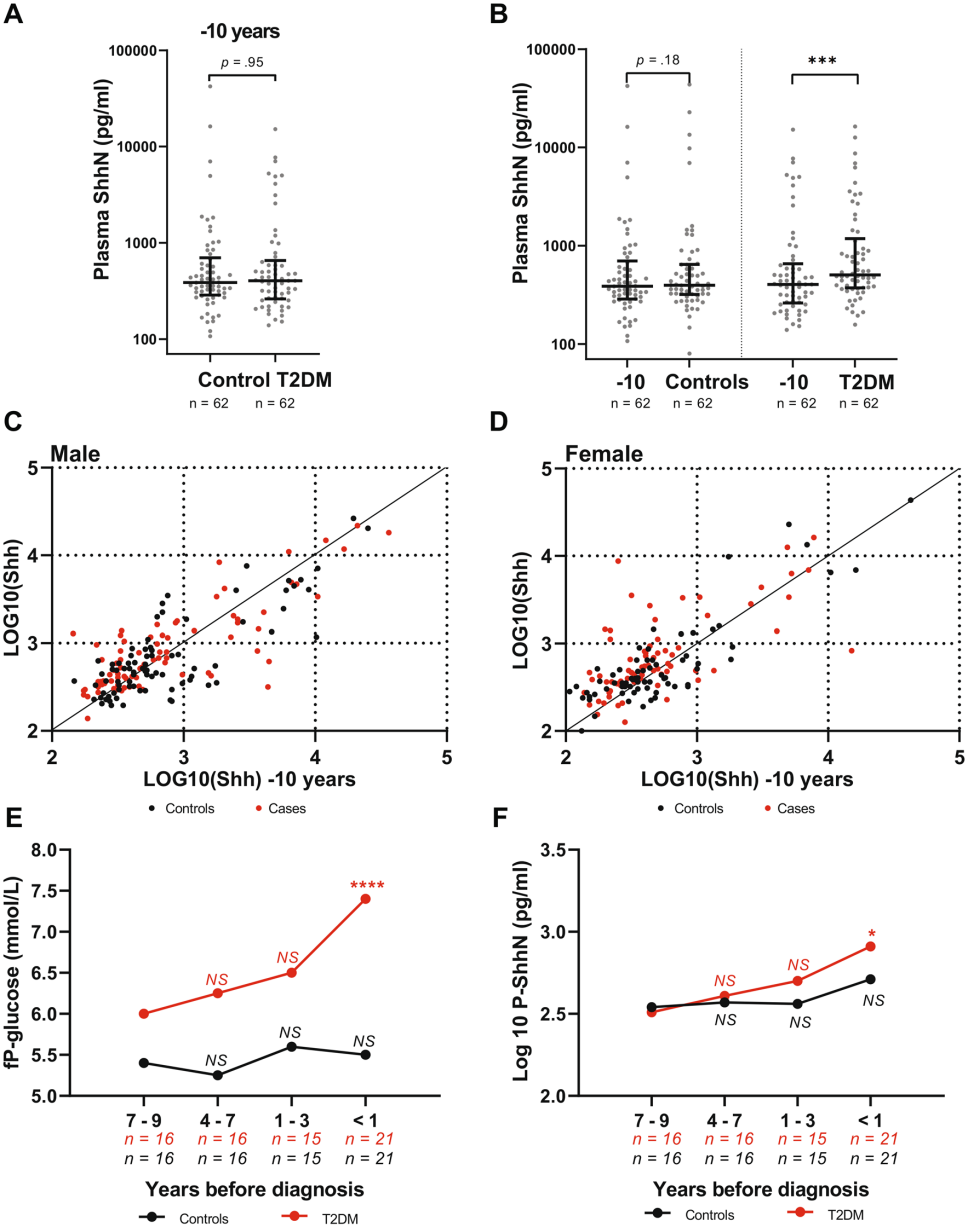
Shh plasma levels increase in parallel with type 2 diabetes development in females. (A) Comparison of −10 years samples of controls and diabetes cases. (B) Comparison of samples taken 10 years (−10 years) prior to the post diagnosis samples analyzed, a significant increase was observed in females with T2DM (*p* = 0.0004). (C) Fasting glucose (adj. *p* < 0.0001) and (D) median Shh levels (adj. *p* = 0.014) in females as diagnosis approached. (E, F) −10 years and the post diagnosis fasting P‐glucose respective Shh level plotted for females. Statistical analyses included Wilcoxon signed‐rank, Mann–Whitney *U*, and Kruskal–Wallis tests, with Dunn's post hoc correction. NS = not significant; *adj. *p* < 0.05; ***adj. *p* < 0.001.

In the VIP case–control study, all individuals donated two samples separated by 10 years. Shh levels in the first samples did not differ between the control and diabetic cases (Figure 6A) for men (*p* = 0.89) or for women (*p* = 0.95). Interestingly, however, there were no significant changes in Shh level over 10 years for control men (*p* = 0.62) or women (*p* = 0.18). Thus, despite the large inter‐individual variability, Shh levels were stable in individual subjects over time (Figure 6B).

Consistent with an association with T2DM progression, female diabetic cases showed an increase in plasma Shh levels over 10 years (*p* = 0.0004) (Figure 6B). For men who developed T2DM, we did observe a trend toward higher Shh levels in the second sample, but it was not significant (*p* = 0.27). When we plotted Shh values from first versus second samples (Figure 6C), only a subset of female T2DM cases showed a large increase in Shh levels from pre‐ to post‐diagnosis timepoints (Figure 6C). The individuals with the most prominent change showed an initial low baseline Shh level and shifted to mid‐range but not extreme Shh levels (Figure 6C). Female diabetics were also clearly distinguished by their lower correlation coefficient and lower intraclass coefficients (Table S5). This shift was absent in males (Figure 6D).

To gain temporal resolution on the increase in Shh levels in women, we calculated the time to diabetes diagnosis by taking the difference between age at diagnosis and age at sampling. To visualize Shh progression, we divided the ‘time to diagnosis’ variable into bins. As expected, we saw a clear increase in fasting glucose as cases approached diagnosis (Figure 6E). Similarly, there was a positive linear trend in Shh levels over time in diabetic women (*p* = 0.01). For diabetes cases, with ‘7–9 years before diagnosis’ as a reference, Shh levels became significant only within 1 year before diagnosis (adj. *p* = 0.014) (Figure 6F).

Collectively, these findings demonstrate that plasma Shh levels are longitudinally stable in healthy individuals but rise in a sub‐set of females shortly before T2DM diagnosis. This pattern suggests that Shh may serve as an early biomarker of long‐term glycemic deterioration and T2DM onset in females.

### 3.7 Shh Is Expressed and Secreted From Human Enterocytes in Response to Glucose and Insulin

*Drosophila* have Hh levels, which are set early in life and stable for most of life [9], indicating that systemic Hh regulation might be a conserved feature. In *Drosophila*, the sugar induced systemic Hh originates from the midgut [9]. Shh is also expressed in the enterocytes of the small intestine in leeches [29], chickens [30] and mammals, including humans [14, 15], suggesting that the intestinal epithelium may serve as an ancestral source of systemic Shh in response to nutritional signals.

**FIGURE 7:**
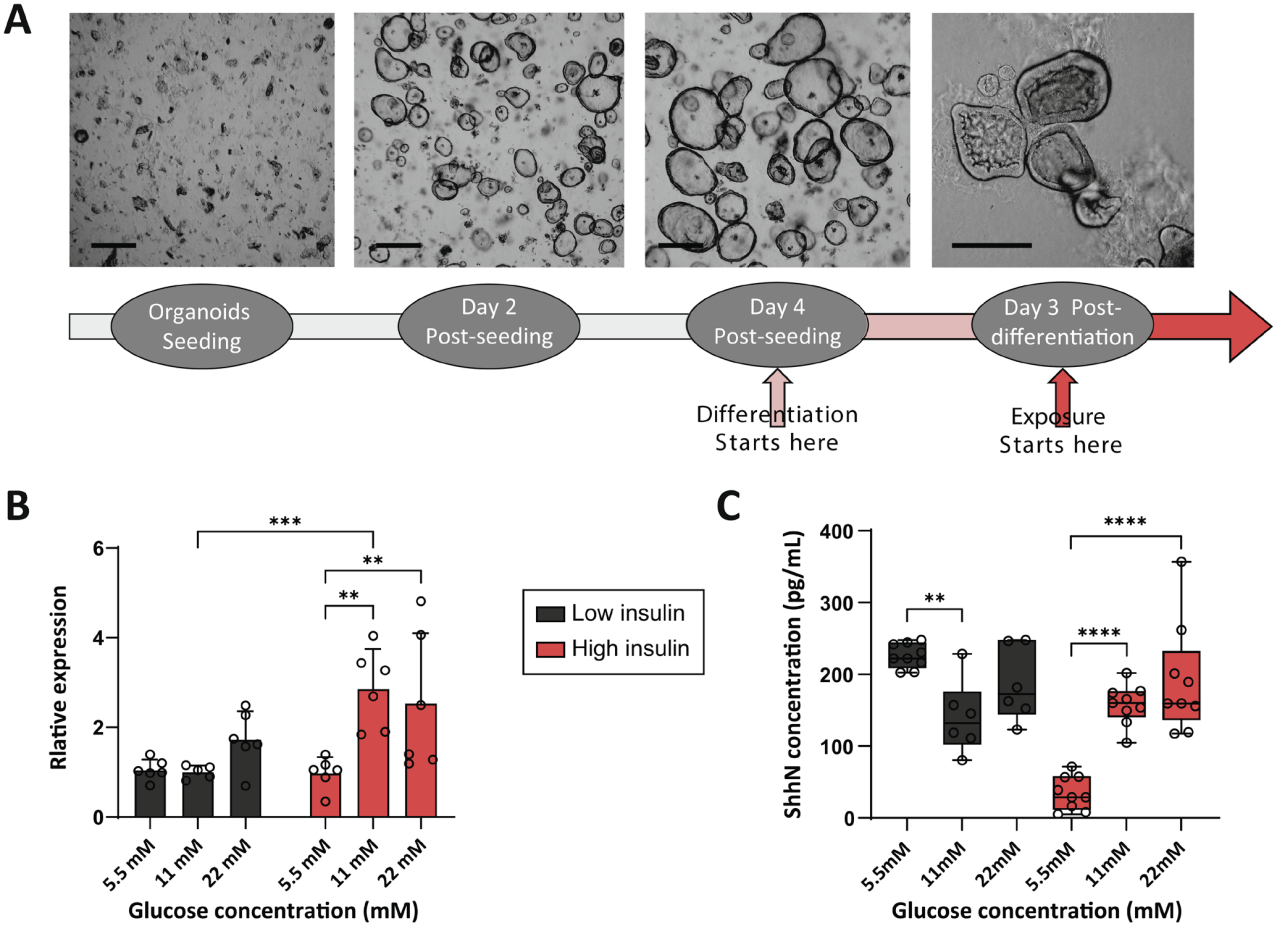
Intestinal organoids express Shh in response to insulin and glucose. (A) Experimental timeline and representative brightfield micrographs showing morphological development of human intestinal organoids over time (scale bars = 200 μm). Differentiation was initiated on day 4 post‐seeding, and treatment/exposure began on day 3 post‐differentiation. (B) Relative expression of Shh in human intestinal organoids exposed to different glucose concentrations under low (0.1 nM) and high (1.7 μM) insulin levels. Data are normalized to 5.5 mM glucose, 0.1 nM insulin. (C) SHH ligand (ShhN) secretion levels in culture media under varying glucose and insulin conditions.

To test this hypothesis, we used human small‐intestinal organoids, derived from crypt stem cells (Figure 7A), which recapitulate the structure and function of the intestinal epithelium. Quantitative PCR analysis revealed that under fasting conditions (5.5 mM glucose and 0.1 nM insulin), organoids expressed basal levels of Shh (Figure 7B). When we increased insulin levels in the culture medium, Shh expression was unaffected (Figure 7B), suggesting that insulin alone is not sufficient to induce Shh. However, when glucose levels were elevated in the presence of high insulin, Shh expression increased tenfold (Figure 7B), demonstrating that the concerted action of glucose and insulin is required to induce Shh expression in the intestinal epithelium.

To determine whether the increased transcription resulted in enhanced secretion, we quantified Shh protein levels in the culture medium. Consistent with the expression data, Shh secretion was enhanced under high glucose and high insulin conditions, but not when only insulin was elevated (Figure 7C). These results show that Shh expression and secretion in human intestinal epithelium are co‐regulated by glucose and insulin, thus pinpointing the human intestinal epithelium as a plausible source for the glucose‐regulated systemic Shh.

## 4 Discussion

Hedgehog proteins are found in the plasma of flies and humans alike [8, 9, 10]. In flies, sugar regulates systemic Hh level [9] and regulates lipid metabolism [31]. Here, we identify in a large‐scale population screen that elevated Shh levels in human plasma relate to hypertension and T2DM and that this occurs only in females. Both conditions exhibit well‐documented sex differences in pathophysiology and are tightly linked to malnutrition [32, 33]. By combining population screening with case–control validation and mechanistic studies using human intestinal organoids, we further demonstrate that circulating Shh levels, depending on sex, correlate with insulin resistance, hyperglycemia, and T2DM.

Consistent with previous observations [26], healthy males in both cohorts exhibited higher baseline plasma Shh concentrations than healthy females. We also found that elevated Shh levels in males are associated with obesity and increased waist circumference, associations that are weaker (for BMI) or absent (for waist circumference) in females. In contrast, among females, plasma Shh levels rise before T2DM diagnosis and correlate with late‐stage prediabetes markers [27], including reduced β‐cell function (HOMA2‐%B) and elevated fasting plasma glucose. The lower baseline Shh levels in females, combined with a delayed but pronounced increase during late prediabetes, suggest a sex‐specific regulation, potentially reflecting a higher threshold or slower release of Shh in response to glucose dysregulation in females.

We further find a remarkably large inter‐individual variability and longitudinal stability in Shh plasma levels. Although levels varied by approximately two orders of magnitude between individuals in the VIP cohort, more than 80% of males and 90% of healthy females maintained similar Shh concentrations over a 10‐year period. This remarkable temporal stability implies that circulating Shh levels change slowly and may represent a long‐term record of metabolic history rather than reflecting acute nutritional states. Notably, a distinct subgroup of females with initially low Shh levels exhibited a marked increase preceding diabetes onset, suggesting that Shh dysregulation may reflect a defining metabolic transition in the development of T2DM among females.

Extremely high Shh levels were also detected in individuals without direct association with T2DM in both cohorts. These individuals displayed longitudinal stability comparable to those with lower Shh concentrations, suggesting that the elevated levels are not acutely harmful and that the biological activity of circulating Shh may not directly correspond to its total plasma concentration. This pattern is reminiscent of leptin signaling, which also exhibits wide inter‐individual variability but is tightly balanced by negative feedback regulation [34]. Similarly, multiple negative feedback loops constrain the Hedgehog pathway [35], potentially rendering circulating Shh functionally inert above a physiological threshold. A comparable regulatory pattern has been described in Drosophila [9], where individual Hh levels are established early in life and remain stable throughout adulthood. Consistent with this evolutionary parallel, our intestinal organoid studies demonstrate that, as in flies [9], the human small intestine secretes Shh. Neither glucose nor insulin alone was sufficient to induce secretion. Only when both were elevated simultaneously did Shh release increase significantly. Together, these observations support the notion that intestinal Shh secretion represents an evolutionarily conserved mechanism that responds to early glucose dysregulation.

Shh is one of three Hedgehog ligands in humans, whereas *Drosophila* possesses only a single ligand, suggesting that vertebrate Hedgehog signaling has undergone substantial diversification and may exhibit more complex tissue‐specific regulation. Braune et al. [36], report that in humans, adipose tissue expression of DHH and serum IHH decreases with obesity and type 2 diabetes. Also, Shh has been implicated in mice adipose tissue development [37], metabolism [38], and in obese mice upregulated in subcutaneous fat [39]. Shh further regulates the developmental transition to brown fat [40] and local adipose inflammation regulation [12, 41], indicating that ligand‐specific contributions modulate the local signaling in adipose tissue. Thus, our results highlight the importance of determining how systemic Shh regulates and possibly balances the local Shh/Ihh and Dhh signaling in metabolic homeostasis and disease.

Systemic Shh further binds to LDL particles [8, 10, 11] and to inhibitory proteins such as Hedgehog‐Interacting Proteins (HHIP and HHIPL1 and 2) [42, 43, 44, 45], which may act as reservoirs or regulators of Shh bioavailability. Elevated HHIP expression is associated with pancreatitis [46], insulin secretion deviations [47], and to T2DM sex differences [48, 49]. These observations raise the possibility that sex‐dependent regulation of Shh‐binding proteins contributes to the observed sexual dimorphism in Shh signaling and highlight the need to clarify the clinical significance of extreme Shh levels.

Notably, the observed associations between circulating Shh and glucose metabolism may be underestimated, as many participants with diabetes were receiving glucose‐lowering therapy, which could influence Shh levels. In addition, a larger female cohort would be essential to fully characterize the subgroup of females showing the most pronounced Shh changes as our sample size was insufficient to elucidate the mechanism behind this intriguing pattern.

In conclusion, our findings identify circulating Shh as a stable, sex‐dependent sugar metabolism signal that links intestinal function to systemic glucose regulation. In females, elevated plasma Shh levels associate with insulin resistance and T2DM, whereas baseline levels are higher and more metabolically linked to adiposity in males. The requirement of combined glucose and insulin for intestinal Shh secretion, along with evolutionary parallels in *Drosophila*, points to a conserved mechanism responsive to early glucose metabolic imbalance. Additionally, the interplay between Shh, its binding partners, and regulatory feedback loops influences signaling activity and may be disease trajectory. These findings underscore the potential of Shh as a biomarker for early metabolic dysregulation and highlight the need for further investigation into its clinical relevance, especially in females.

## Author Contributions

J.A. performed the Elisa analysis and the statistics. N.T. performed the organoid culture experiments. J.K. and E.C.J. made the expression analysis. N.D. Elisa analysis of the organoid supernatants. J.A. arranged the figures. S.A.L., I.H. and O.R. assisted with the statistical analysis. H.K. supervised and designed the study. M.A. wrote the paper with contributions from J.A., V.M.L., I.B. and H.K.; M.A. conceived, designed, and supervised the study.

## Funding

This work was supported by the Swedish Research Council, grant (2016‐05208 and 2023‐04964) and N.D. was supported by the Kempe Foundation grants (SMK‐1764 and JCK‐3158). N.T., J.K. and V.M.L. was supported by the ERC Consolidator Grant 3DMASH (101170408), the Swedish Research Council (2021‐02801, 2023‐03015 and 2024‐03401), the Novo Nordisk Foundation (NNF23OC0085944 and NNF23OC0084420), the SciLifeLab and Wallenberg National Program for Data‐Driven Life Science (WASPDDLS22:006) and the Robert Bosch Foundation, Stuttgart, Germany.

## Ethics Statement

Approved ethical permits by the Swedish Ethics review Board; The Västerbotten Intervention Programme (VIP) [21] main application Dnr 2013/414‐31 and additional application; Shh analyses Dnr: 2018‐503‐32. The Glass Works Cohort [17] main application Dnr 2009/138‐31 and the organoid application Dnr: 2023–01524‐01 and handling of patient‐derived cells Dnr: 2024‐05808‐01.

## Conflicts of Interest

V.M.L. is CEO and shareholder of HepaPredict AB, as well as co‐founder and shareholder of Shanghai Hepo Biotechnology Ltd. The other authors declare no conflicts of interest.

## Supporting information


**Table S1:** Baseline characteristics of the Glasriket cohort (*n* = 744). Demographic and clinical variables including sex distribution, age, BMI categories, smoking status, and prevalence of chronic diseases. Data are shown as mean ± SD, median (range), or number and percent.
**Table S2:** Descriptive and Mann–Whitney comparisons between sex stratified healthy participants and disease groups in the Glasriket cohort. For each condition, the minimum, 25th percentile, median, 75th percentile, and maximum values are reported together with unadjusted/adjusted *p*‐values, Mann–Whitney *U* statistics, and rank sums. Healthy individuals serve as the reference group.
**Table S3:** Characteristics of the VIP diabetes case–control two‐sample longitudinal cohort. Participant numbers, age, BMI at Sample 1 and Sample 2, and diabetes status relative to sampling are presented for males and females. Treatment categories at the second sampling are listed for individuals with diabetes.
**Table S4:** Metabolic and anthropometric measurements in the VIP study cohort. Fasting plasma glucose, insulin, C‐peptide, HOMA2 %B, HOMA2 %S, HOMA2‐IR, BMI, and waist circumference are reported as mean ± SD together with valid sample counts for males and females in case and control groups.
**Table S5:** Intraclass correlation coefficients (ICC) and Pearson correlation coefficients for repeated measures in males and females. Two‐way mixed‐effects ICCs (absolute agreement, average measures) with 95% confidence intervals are shown for cases and controls, together with corresponding Pearson correlation coefficients and significance values.

## Data Availability

Privacy/ethical restrictions.
